# Development of Two-Dimensional Functional Nanomaterials for Biosensor Applications: Opportunities, Challenges, and Future Prospects

**DOI:** 10.3390/nano13091520

**Published:** 2023-04-29

**Authors:** Shamsa Kizhepat, Akash S. Rasal, Jia-Yaw Chang, Hui-Fen Wu

**Affiliations:** 1 Department of Chemistry, National Sun Yat-Sen University, Kaohsiung, 70, Lien-Hai Road, Kaohsiung 80424, Taiwan; 2 Department of Chemical Engineering, National Taiwan University of Science and Technology, Taipei 10607, Taiwan; 3 School of Pharmacy, College of Pharmacy, Kaohsiung Medical University, Kaohsiung 80708, Taiwan

**Keywords:** 2D nanomaterial, Janus nanoparticles, electrochemical, optical, piezoelectric biosensors

## Abstract

New possibilities for the development of biosensors that are ready to be implemented in the field have emerged thanks to the recent progress of functional nanomaterials and the careful engineering of nanostructures. Two-dimensional (2D) nanomaterials have exceptional physical, chemical, highly anisotropic, chemically active, and mechanical capabilities due to their ultra-thin structures. The diversity of the high surface area, layered topologies, and porosity found in 2D nanomaterials makes them amenable to being engineered with surface characteristics that make it possible for targeted identification. By integrating the distinctive features of several varieties of nanostructures and employing them as scaffolds for bimolecular assemblies, biosensing platforms with improved reliability, selectivity, and sensitivity for the identification of a plethora of analytes can be developed. In this review, we compile a number of approaches to using 2D nanomaterials for biomolecule detection. Subsequently, we summarize the advantages and disadvantages of using 2D nanomaterials in biosensing. Finally, both the opportunities and the challenges that exist within this potentially fruitful subject are discussed. This review will assist readers in understanding the synthesis of 2D nanomaterials, their alteration by enzymes and composite materials, and the implementation of 2D material-based biosensors for efficient bioanalysis and disease diagnosis.

## 1. Introduction

In response to a growing demand for the detection of biomolecules with sizes on the micrometer scale, including nucleic acids, proteins, and others [1], biosensors have quickly developed into powerful diagnostic and therapeutic tools [2,3,4]. Biological biosensors can also be used to find microbes such as bacteria, viruses, and pathogens. Utilizing biosensors, it is possible to find organisms such as viruses, pathogens, and bacteria [5]. Numerous biological substances, including DNA [6,7], RNA [8], dopamine [9,10], uric acid [11], viruses [12], glucose [13], and others, can be detected by biosensors. As a result, biosensors are used in a wide range of applications, such as healthcare, pharmacy, and biomedicine [14,15,16,17]. These biosensors fall into a number of categories, including electrochemical biosensors [18], bioluminescent biosensors [19], optical biosensors [20,21], and mass-based biosensors [22]. By utilizing their distinct chemical and physical properties to enhance analytical performance, numerous nanomaterials have been developed for use in biosensor construction. When analytes are extracted from living systems, it is typical for their concentration to be quite low. This is particularly valid in the case of biomarkers linked to specific diseases. Due to this, an enormous amount of time and effort has been put into developing and designing ultrasensitive bioassays over the past few decades. A key method for improving the detection signal in biosensors is the use of the proper tools, which can be achieved by building intricate instruments and performing intricate detection procedures. Biosensors’ capacity to detect biological molecules has been demonstrated to be directly influenced by the composition, shape, architecture, and physicochemical characteristics of the exploited nanostructures [23]. The use of nanostructures with particular characteristics, such as high conductivity, high surface area, and a high level of biological compatibility, can be advantageous for biosensors. The identification of novel advanced nanomaterials is crucial for developing biosensors with good accuracy, high sensitivity, and a relatively low detection limit [24]. Finding new, useful nanostructures is one approach to achieving this objective.

**Figure 1:**
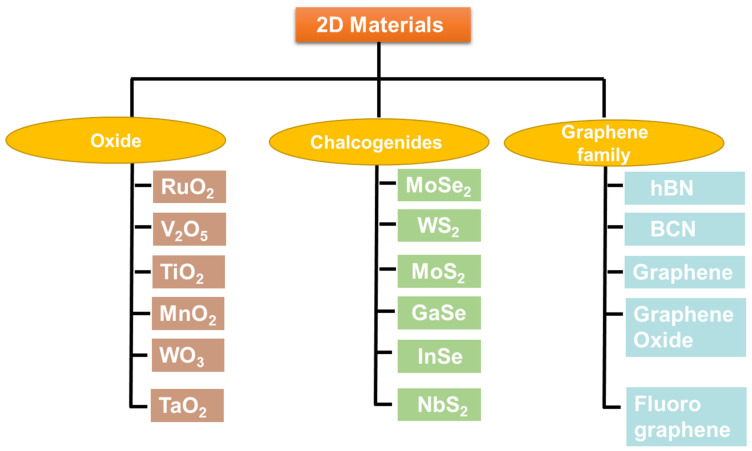
Schematic representations of the key 2D nanomaterials used in biosensor applications.

Due to the astounding physicochemical properties that 2D layered nanomaterials display, interest in this field has exploded recently [25,26]. Studying the characteristics that set 2D nanomaterials apart from their bulk counterparts is most fascinating. Various 2D nanomaterials (Figure 1) have been widely used for manufacturing numerous biosensors due to their distinctive structure and properties, which include high sensitivity, biocompatibility, chemical stability, mechanical stability, easy functionalization, and quick response time [27]. Due to their potential to offer an astonishingly high density of active surface sites over a sizable area, 2D nanomaterials are advantageous for biological sensing applications [28]. The 2D material family also stands out because it has the potential to display a variety of electrical properties, such as insulating, conducting, and semiconducting, as well as being metallic and semi-metallic [29]. The ability to quench or emit fluorescence, plasmonic activity, and a variety of other optical phenomena are just a few of the optoelectronic properties they exhibit [30]. Two-dimensional nanomaterials can be designed to respond specifically to a limited range of analytes by functionalizing or incorporating defects into their edges [31]. Two-dimensional nanomaterials have the potential to develop into nanostructures for the upcoming generation of diagnostics and biosensors because of their unusual properties, layer-dependent band structure, and heterostructures.

The current review article aims to give readers an overview of recent advancements in 2D nanomaterials that can be used in biosensing applications. This review will start by giving a brief overview of the various synthesis techniques, including top-down and bottom-up approaches, that can be used to produce 2D nanomaterials. After that, we focus on the numerous types of biosensors that are used as nanomaterials in biosensing applications and outline them with examples and illustrations. The development of numerous 2D nanomaterial-based biosensors will then be the main focus of our review. We conclude by talking about some exceptional 2D nanomaterials that can be used in biosensing. The presentation concludes with a glance into the near-future in an effort to inspire more fascinating studies in the not-too-distant future. After briefly outlining the outlook and conclusion, we list the present advantages and disadvantages of next-generation biosensors.

## 2. Preparation of 2D Nanomaterials

In the fabrication of 2D nanomaterials, a variety of synthesis methods have been reported. Exfoliation of a bulk nanomaterial can result in the formation of single or multi-layer nanosheets, with the exfoliated layers adhering to one another due to weak Van der Waals interactions [32]. These weak connections disintegrate, leading to the formation of 2D nanomaterials. It is possible to use both top-down and bottom-up strategies, with the latter suggesting that a compound is built from fundamental building blocks [33]. Bottom-up approaches include methods such as wet chemical synthesis [34,35], microwave-assisted chemistry [36,37,38,39], and ultrasound probe synthesis [40,41], among others. Under the top-down category of exfoliation techniques, mechanical exfoliation [42], direct liquid exfoliation [43], ultrasonic exfoliation, and chemical oxidation exfoliation [44] are all included. The fabrication of 2D nanomaterials for biosensing applications is covered in the section that follows, along with a few examples of fabrication that are relevant.

### 2.1. Top-Down Approach

#### 2.1.1. Mechanical Exfoliation

The layers are peeled away from the bulk material using mechanical energy. Due to Van der Waals forces, the interaction between the layers in these layered nanomaterials is weak despite the strong chemical bonds that exist in the planes between the layers. As part of the mechanical exfoliation procedure, adhesive Scotch tape is used to peel the layer away from the bulk crystal [42]. Among other benefits, it produces a simple design, a large lateral size, and a high-quality material yield [45]. For use in electrochemiluminescent biosensing, a monolayer was created from bulk 2D graphitic carbon nitride. In biosensing applications, carbon nitride (CN) has been widely used. The responsiveness limits and its applicability were nevertheless constrained. A modification of the interfacial was then found to resolve this issue. The idea was then put forth to use mechanical exfoliation to produce carbon nitride nanosheets with improved bulk material functionality. This mechanism enables the efficient conjugation of a variety of biomolecules via various chemical interactions. In this instance, exfoliated carbon nitride nanosheets were used to immobilize DNA probes, improving the electrochemiluminescence sensing performance. Therefore, the physical adsorption mechanism was less effective than the method of interfacial modification through mechanical exfoliation for improving sensing performance. The low yield and lack of significant output are two additional drawbacks [46] that restrict the use of mechanical exfoliation methods in the manufacturing industry.

#### 2.1.2. Ultrasonic Exfoliation

Ultrasonic exfoliation is one of the most effective methods for peeling off a thin layer from a larger piece of material. In comparison to mechanical exfoliation, requires less effort and a more relaxed approach. An effective surfactant is required for the ultrasonication process. However, the quality of the end result depends on how long you sonicate for and what kind of solvents you use. Graphene exfoliation in water has been attempted in a variety of ways recently [47]. The bulk material was used to create a single layer of graphene. Graphene is typically extracted by “liquid exfoliation”, or the direct sonication of bulk crystals in a solvent or with surfactants [48,49]. Graphite can be exfoliated to create bi-layer graphene oxide (GO) [50] in a manner analogous to that used to transform it into single-layer GO via ultrasonic treatment. GO was created by subjecting graphite to ultrasonic waves at 40 °C and 150 W for 30 min. The use of a strong oxidizing agent was required in order to obtain a monolayer product via ultrasonic exfoliation. To get GO out of graphite, it was heated to 30 °C with a strong oxidizing agent for 25 min. In a different study, graphite was ultrasonically treated at 50 °C for 15 min to create reduced graphene oxide (rGO), which also did not employ an oxidizing agent. The solution’s processability, low cost, and simplicity make ultrasonic exfoliation preferable to mechanical exfoliation for large-scale production. There are a few problems with this method, however: a low single-layer yield, shallow layers, and an unpredictable layer morphology [51,52,53].

#### 2.1.3. Ion-Change Exfoliation

This method can be used to exfoliate layered 2D nanomaterials that have strong ionic connections between their layers. For strong ionic solids, the layer separation step is the most difficult part of this process [54]. One possible medium for carrying out the reaction is hydrochloric acid (HCl), for instance. Exfoliation of cobalt oxide occurs as a result of the penetration of tetrabutylammonium (NBu_4_)^+^ into the layers of the cobalt oxide [55,56]. This method demonstrates solution processability, impressive monolayer yields, as well as fast production speeds. On the other hand, when exposed to the oxygen and moisture that are present in the air, this method turns into an extremely delicate one. In addition to this, the monolayers with larger flaws are subsequently produced after the initial ones [57,58,59,60,61].

### 2.2. Bottom-Up Approach

For the production of high-quality nanosheets with large lateral dimensions, these exfoliation techniques are by far the most effective option. The previously mentioned synthesis technologies have a limited amount of applicability because of the poor scale of manufacturing that they utilize [62]. As a result, they are not applicable to production on a massive scale at industrial levels. The atomic level is the starting point for bottom-up approaches, which use self-assembly to construct a robust nanostructure framework. Here, we will take a glance at a small subset of the bottom-up techniques that go into the production of 2D nanomaterials.

#### 2.2.1. Chemical Vapor Deposition (CVD) Method

When it comes to mass-produced 2D materials, the CVD method is state-of-the-art. At elevated temperatures, a volatile precursor is introduced to the substrate. Due to its complicated setup, high pressure, and extremely high temperature, it is not feasible for widespread use. It is possible to create 2D material by starting the reaction and letting the precursors settle onto a wafer. Few-layered 2D nanomaterials with high performance and quality can be produced using CVD [63]. In order to mass-produce copper substrates on which graphene has been deposited, a team of researchers led by Xuesong Li and colleagues developed a method [64]. The CVD method of depositing graphene nanosheets results in a uniformly thick coating. The CVD method is used to produce graphene on copper foil at a temperature of 1000 °C. Graphene with consistent layers is produced when copper is added to the mixture. The volatile precursors play a crucial function by accelerating the breakdown of the volatile substrate at high temperatures and vacuum states, resulting in 2D crystals [65,66]. The prepared layer was high-quality, extremely deep and wide, and easily modifiable. High-temperature and low-pressure systems are the main obstacles that make the technique arrangement complicated and energy-consuming [67,68,69].

#### 2.2.2. Wet Chemical Method

Wet chemical synthesis employs an aqueous solution in the presence of surfactants to directly produce the desired product. Its high yield, huge manufacturing potential, and solution processibility are all advantages; however, creating a single-layer nanosheet that is uniform throughout its whole surface can be difficult using this approach. This technique is widely employed in the production of flat materials. Some other methods that fit into this category are hydrothermal synthesis, solvothermal synthesis, and template synthesis [70,71,72,73,74,75,76,77].

#### 2.2.3. Hydrothermal Method

Hydrothermal or solvothermal synthesis governs the production of 2D nanomaterials. It promotes chemical reactions within a specific temperature range and a longer reaction time. It has been widely adopted because of the simplicity of the method and the high quality of the byproduct. For instance, hydrothermal synthesis of tungsten trioxide (WO_3_) was reported without the addition of surfactant [78]. The reaction was carried out in an acidic medium to speed up the process. The precursor, 3 mmol of sodium tungstate, was first dissolved in 20 mL of distilled water and mixed carefully. After waiting for 40 min, 10 mL of diluted HCl was added, and the mixture was agitated. Then, the mixture was kept in the autoclave for 24 h at 200 °C. Large-scale 2D nanocubes of WO_3_ were produced after completion of the reaction. Several types of analyzing techniques were used to confirm the morphological and optical properties: X-Ray diffraction analysis (XRD), photoluminescence (PL), scanning electron microscopy (SEM), UV- and visible spectroscopy. The resulting 2D WO_3_ nanocubes exhibited favorable shape and optical characteristics. Both reaction time and temperature played crucial roles in determining the final morphology of the product. Hydrothermal hybridization is a common method of large-scale production [79,80,81,82].

#### 2.2.4. Microwave-Assisted Method

Compared to other techniques such as hydrothermal and sonication, this process has a simple procedure and a fast reaction time. Moreover, the microwave approach is scalable, efficient, and inexpensive [83,84,85]. A reduction in response time, high product purity, and regulated particle size and morphology are all possible outcomes [86]. Moreover, in recent times, the microwave technique has been used to develop Ni-based hydroxide, Ni(OH)_2_ [87]. Similarly, microwave-assisted synthesis of Ni(OH)_2_ nanoplatelet/electrospun carbon nanofiber (ECF) was conducted for the sensing of glucose substrate [88]. Effective microwave-assisted synthesis was used for the in situ development of crystalline Ni(OH)_2_ nanoplatelets to generate Ni(OH)_2_/ECF hybrids. Ni(OH)_2_ nanoplatelets with thicknesses between 60 and 150 nm were evenly dispersed across the ECF surface, preventing the nanoparticles from clumping together. Optimal results were achieved after 20 min of microwave irradiation at 90 °C. In order to detect Hg^2+^ ions via fluorescence biosensing, a simple and efficient microwave method was used to synthesize chromium oxide nanoparticles (Cr_2_O_3_ NPs) [89]. In the reaction, a couple of metal ions (Mn^2+^, Cr^3+^, Zn^2+^, Cu^2+^, and Ni^2+^) and sodium citrate as oxidizing agents were heated using the microwave. Microwave heating then triggered the reaction of Cr^3+^ and sodium citrate to form the colloids; owing to the Tyndall effect, only Cr^3+^ was efficiently produced after heating, and a blue-green precipitate was obtained after centrifugation. In line with this discussion, microwave irradiation techniques are essential in the fabrication of colloidal nanoparticles. The absence of the above events in the mixed solution heated just conventionally demonstrates that microwave heating is essential for the development of colloids.

## 3. Types of Biosensors

**Figure 2:**
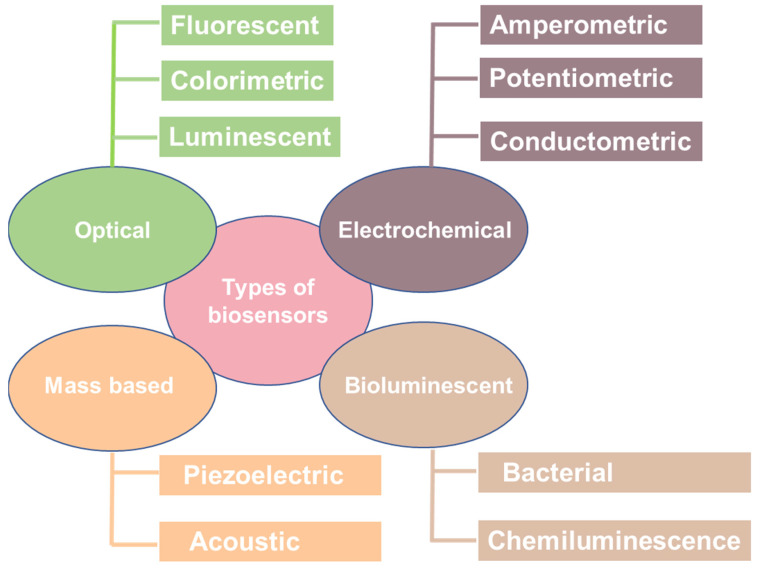
A schematic illustrating classification of biosensors.

A biosensor is an analytical tool that makes use of biologically distinct detecting elements and transducers [90,91,92]. Recent examples of sensing elements include enzymes [93,94], tissues [95,96], antibodies [97,98], and microorganisms [99,100]. Based on the underlying transducer technology, four distinct families of biosensors have emerged. The most common types of biosensors include those that rely on electricity (electrochemical) [101,102], light (optical) [103,104], pressure (piezoelectric) [105,106], or heat (calorimetric) [107,108] potentiometric molecules. Conductometric [109,110], potentiometric [111,112], and amperometric biosensors [113,114] are all categorized under electrochemical biosensors. Different forms of biosensors such as interferometric biosensors [115,116], fluorescent biosensors [117,118], and luminescent biosensors [119,120] are included in optical biosensors. In another case, acoustic [121,122] and ultrasonic biosensors [121] are discussed in the piezoelectric section. Figure 2 provides a comprehensive breakdown of the various biosensor classifications.

### 3.1. Electrochemical Biosensors

The electrochemical transducer is a crucial component of many biosensors, making electrochemical sensors among the most popular types. Both a three-electrode and a two-electrode setup are frequently used. The working, reference, and counter electrodes make up the three-electrode systems. It has the ability to distinguish between living and non-living things. The total sensing process is significantly influenced by the working electrode’s surface. For instance, the reference and counter electrodes of the three-electrode configuration are, respectively, made of Ag/AgCl and platinum (Pt). The chemical signal is then progressively converted into an electrical signal. However, the experiment’s solution may have an impact on the sensor device’s performance and overall results [123,124].

#### 3.1.1. Amperometric Biosensors

This is one type of electrochemical sensor that has found widespread use. In order to deduce the mechanism used by a redox reaction, it is necessary to establish the linear relationship between the measured current and the analyte concentration gained from the reaction. In this case, the analyte concentration will have a linear relationship with the intensity of the current show. Electrons are constantly moving from one molecule to another as they do so. Additionally, it helps electrons travel more quickly between locations. Amperometric biosensors work by oxidizing or reducing the target analyte at the electrode’s surface to generate a current whose magnitude is proportional to the analyte’s concentration. The current is then measured using a device called a potentiostat, which applies a voltage to the electrode and records the response. It is crucial for a mediator to have rapid reactivity with the targeted molecule, stability, and reversible heterogeneous kinetics. It has been significantly used in glucose monitors, which are used by diabetic patients for keeping track [125,126].

#### 3.1.2. Potentiometric Biosensors

In order to measure the shifts in ionic concentration, potentiometric biosensors with electrodes that selectively react to specific ions have been applied. Ion activity and selectivity that occur during the reaction might be gleaned from this value [127]. In the absence of current flow, it monitors the potential between the working electrode and the reference electrode [128]. Field-effect transistors (FETs) form the basis of many potentiometric devices. It is commonly used to detect changes in pH, ion concentrations, and the rate of enzyme-catalyzed biocatalytic reactions [129].

#### 3.1.3. Conductometric Biosensors

It relies, as the name suggests, on measuring the electrical resistance between a pair of electrodes. Enzymes and electrical conductivity sensors share the same source. Changes in ionic strength and conductivity occur in a solution between electrodes when an enzyme reacts with it. Chemical processes that cause a change in a solution’s concentration can be studied with conductometric devices [130,131].

### 3.2. Optical Biosensors

Enzymes or fluorescent dyes conjugated at one end of an optical fiber make up an optical biosensor. The reagent at the end of the optical cable undergoes an interaction with the light. The reflected light is then collected, and the results are analyzed. The amount of analyte can be deduced from the brightness of the reflected light. Here, we will break down the optical sensors into their respective subtypes [132].

#### 3.2.1. Luminescent Biosensors

Luminescent molecules are responsible for the absorption and emission of light. When photons collide with molecules in their ground state, the molecules undergo absorption of light and are excited to a higher energy level. The emitted electrons are then transported to a lower energy level by emitting light energy. Several unique, bright biosensors have been recently reported by different researchers. The energy level of the system determines how the molecules are stimulated. In the process of chemiluminescence, for example, light is emitted as a byproduct of chemical reactions. Bioluminescence is caused by living things and the chemical processes that they go through [133].

#### 3.2.2. Fluorescent Biosensors

A fluorescent biosensor is highly sensitive and selective as compared to other biosensing devices. Additionally, the short response time of the fluorescent biosensor is another advantage that is usually preferable in clinical diagnostics. Briefly, the sensor device absorbs photons at lower wavelengths and emit in longer wavelengths, with the phenomenal time estimated to be 109 to 108 s. Intermolecular charge transfer (ICT) and Fluorescence Resonance Energy Transfer (FRET) are the two widely known components of fluorescent biosensors. The FRET biosensor uses the ratio of two fluorescence intensities to calculate results where it is not affected by environmental variables such as sensor concentration or changes in the excitation source [134,135].

### 3.3. Piezoelectric Biosensors

Antigens in the picogram range can be detected using a piezoelectric immunosensor both in liquid and solid phases. The frequency of the signal shifts when the antigen interacts with the antibody receptor. Hence, the frequency shift is detected by the piezoelectric biosensor. Notably, a piezoelectric biosensor device is extremely sensitive to a change in the mass of the substrate. Moreover, sensing devices have been utilized in different application areas such as the detection of cancer, DNA hybridization, DNA strands, and viral detection [136].

## 4. Two-Dimensional Materials for Biosensing

**Figure 3:**
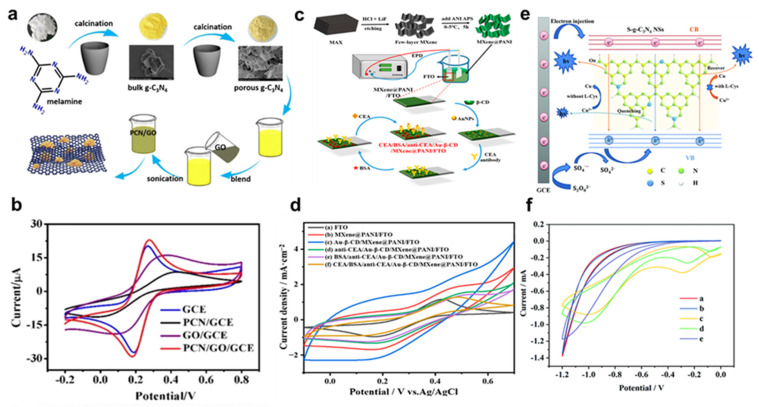
(**a**) Schematic representation of preparation steps involved in PCN/GO. (**b**) Representative cyclic voltammetry curves of GCE, PCN/GCE, GO/GCE and PCN/GO/GCE composites [148], copyright 2020, Springer Nature. (**c**) Synthesis of MXene/PANI and electrochemical immunosensor. (**d**) CV-measured curves of composite for detection of carcinoembryonic antigen [149], copyright 2022, MDPI. (**e**) Mechanism for detection of L-cysteine. (**f**) Cyclic voltammetry behavior of S-g-C_3_N_4_/GCE. (a) absence of Cu^2+^; (b) presence of 100 µm L-cysteine; (c) presence of 50 µm Cu^2+^; (d) presence of 1 µm L-cysteine and 50 µm Cu^2+^; (e) presence of 100 µm L-cysteine and 50 µm Cu^2+^ [150], copyright 2019, Royal Society of Chemistry.

In 2004, scientists discovered the 2D substance graphene [137]. Since its discovery, graphene has been the subject of extensive study and put to use in many different areas, including but not limited to electronics, photonics, materials science, sensing, and even biomedical applications. This is because graphene has exceptional chemical and physical characteristics. It has a high optical and electrochemical efficiency, a low rate of charge recombination, quick carrier transport, a high mechanical strength, and a large surface area [138]. Researchers have been motivated by the intriguing features of 2D nanomaterials to explore new 2D nanomaterials and predict their imminent emergence. As a result, hexagonal boron nitride (hBN) [139], boron-carbon-nitride (BCN) [140], transition metal dichalcogenides [141] (TMDCs: MoS_2_, MoSe_2_, WS_2_, WSe_2_, etc.), transition metal oxides [142,143,144,145,146] (TMOs: LaVO_3_, LaMnO_3_), and others (Li_7_MnP_4_, MnP_4_), as well as layered complex oxides, have been successfully investigated. The unique characteristics of 2D nanomaterials make them promising for use in biosensing. High surface-to-volume ratio, sensitivity, tunable structure, and bandgap are what make 2D nanomaterials so popular in the sensing sector, especially in biosensing [147]. These 2D nanomaterials can be functionalized with various biomolecules, such as antibodies, DNA, and enzymes, to specifically detect target analytes. The large surface area of 2D nanomaterials enables the immobilization of a high density of biomolecules, leading to high sensitivity in biosensing. Moreover, the unique electronic properties of 2D nanomaterials enable label-free detection, where the analyte can be detected directly by changes in the electronic properties of the 2D material upon binding. Overall, 2D nanomaterials show great promise for biosensing applications, and ongoing research is exploring the potential of these nanomaterials in various biosensing platforms. There are some more related studies show in Figure 3 [148,149,150]. Figure 3a shows the schematic representation of the preparation steps involved in PCN/GO. Figure 3b presents the representative cyclic voltammetry (CV) curves of GCE, PCN/GCE, GO/GCE and PCN/GO/GCE composites [148]. Figure 3c demonstrates the synthesis process of the MXene/PANI and electrochemical immunosensor. Figure 3d shows the CV-measured curves of composite for detection of carcinoembryonic antigen [149]. Figure 3e presents the mechanism for detection of L-cysteine. Figure 3f indicates the Cyclic voltammetry behavior of S-g-C_3_N_4_/GCE.

### 4.1. Graphitic Carbon Nitride (g-C_3_N_4_)

g-C_3_N_4_ is a 2D substance with unusual physical and chemical properties that have sparked extensive scientific attention. A bandgap of 2.7 eV, metal-free visible light photocatalyst, simple synthesis route, and strong thermal and chemical stability are all desirable characteristics [151]. g-C_3_N_4_ nanosheets, which are 2D nanomaterials, are widely employed in photoelectrochemical biosensing [152,153]. The g-C_3_N_4_ nanosheets were obtained using a straightforward thermal breakdown process. It is the most straightforward method for mass producing g-C_3_N_4_. Both the amount of nitrogen in the final product and its other characteristics are sensitive to the nature of its precursor. Cyanamide, dicyandiamide, ammonium thiocyanate, urea, and melamine are all suitable as precursors. Since the obtained g-C_3_N_4_ nanosheets have a planar structure and sp^2^-linked carbon and nitrogen, it is extremely stable. The nanosheets were employed as an electrode in a photoelectrochemical biosensor. The advantages of this biosensing method include its inexpensive price, excellent sensitivity and selectivity, low-term stability, and quick response time. The performance of a photoelectrochemical biosensor is enhanced by the use of a g-C_3_N_4_ nanosheet because of its superior photon-harvesting capabilities. With this technique, three electrodes are used. A photosensitive substance is added to the working electrode, with a nanosheet of g-C_3_N_4_ being used as the photosensitive material in this application. Platinum (Pt) and a standard electron are used to construct the counter electrode. The N and C structures in graphene carbon nitride coordinate with metal ions [154].

**Figure 4:**
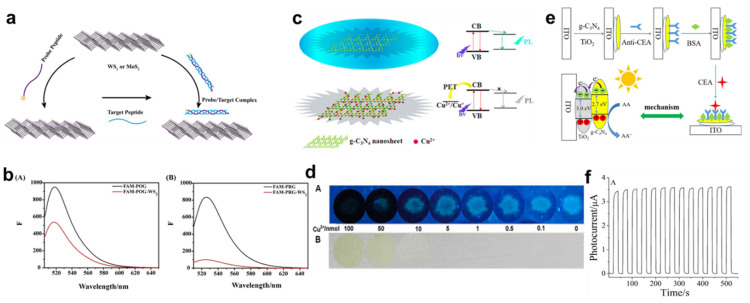
(**a**) Schematic representation of WS_2_ and MoS_2_ biosensor approach. (**b**) Quenching of fluorescence intensity of FAM-POG and FAM-PRG in the presence (red) and absence (black) of WS_2_: (**A**) FAM-POG, (**B**) FAM-PRG [159], copyright 2017, Springer Nature. (**c**) Mechanism of Fluorosensor-based g-C_3_N_4_ for the detection of Cu^2+^ (**d**) Various concentrations of Cu^2+^ under irradiation in the presence of (**A**) sunlight, and (**B**) UV light [155], copyright 2013, American Chemical Society. (**e**) Schematic illustration of PEC immunosensor synthesis steps. (**f**) Immunosensor stability of 550 s under irradiation on/off condition for the selectivity of PEC immunosensor [160], copyright 2016, Springer Nature.

As a result of this property, g-C_3_N_4_ is a promising sensing material. For the detection of alkaline phosphatase, g-C_3_N_4_ nanosheets have been used in previous research. Because of its complex structure, pyrophosphate inhibits the g-C_3_N_4_ fluorosensor for Cu^2+^ coordination to g-C_3_N_4_. Hence, a label-free sensor using pyrophosphate as a substrate was developed. When Cu^2+^ and pyrophosphate are not coordinating with g-C_3_N_4_, the fluorescence will quench as a result of photoinduced electron transfer [155]. Cu^2+^ forms a chelate with pyrophosphate in the presence of pyrophosphate, preventing it from binding with the g-C_3_N_4_ nanosheet. The addition of alkaline phosphatase catalyzes the conversion of pyrophosphate to phosphate, which then forms a weak contact with Cu^2+^. Cu^2+^ will bond to g-C_3_N_4_ in this case. Fluorescence is quenched, demonstrating the presence of active g-C_3_N_4_. In order to increase the sensitivity and selectivity of the devised method for detecting alkaline phosphatase, the use of g-C_3_N_4_ with its enhanced luminous intensity, and photo- and chemical stability was used. However, the rapid recombination of charge carriers in g-C_3_N_4_ limits its performance. Hybridization of g-C_3_N_4_ with other nanomaterials is proposed as a solution to this significant restriction. As a result, photoelectrochemical biosensors use g-C_3_N_4_ hybridized with TiO_2_ [156]. These nanohybrid components were used for glucose detection. Two-dimensional g-C_3_N_4_-TiO_2_ was successfully synthesized. A huge amount of biomolecules can be accumulated on the 2D TiO_2_ nanosheet that was produced. The hybrid was made apparent by using g-C_3_N_4_. The bandgap is reduced as a result of this property. The glucose biosensor demonstrates both great selectivity and sensitivity, and it may be triggered by light in the visible spectrum. Hydrothermal synthesis was used to create the g-C_3_N_4_-TiO_2_ nanosheet. Using heat, this technique is the most straightforward and productive at scale The sample is heated to 200 °C for 12 h. Isopropyl alcohol and diethylenetriamine are the starting materials. For the PEC biosensor, the produced nanohybrid is deposited in the ITO electrode. This shows that g-C_3_N_4_-TiO_2_ significantly enhances PEC biosensing compared to bare g-C_3_N_4_ and TiO_2_. As a means of detecting glutathione, research into fluorescence sensors using g-C_3_N_4_-MnO_2_ nanocomposite was conducted. Glutathione was measured in both liquid and cellular environments. The g-C_3_N_4_ nanosheet had a high surface area, and quantum yield, and emitted a bright fluorescent light. The nanocomposites were produced using a simple one-step redox synthesis process. FRET causes fluorescence quenching when MnO_2_ is deposited on g-C_3_N_4_ [157]. Glutathione works by converting MnO_2_ back into Mn^2+^, which in turn gets rid of FRET and brings g-C_3_N_4_ fluorescence back to normal. In this way, the fluorescence feature of g-C_3_N_4_ is used to identify thione. The study presented here demonstrates the successful application of a straightforward method with little upfront costs, high biocompatibility, fast detection, and low cytotoxicity in living cells. A sulfur-doped graphitic carbon nitride nanosheet (S-g-C_3_N_4_) has been reported to improve electrochemiluminescence performance in the detection of L-cysteine [150]. Trithiocyanuric acid was employed as a precursor in an in situ sample preparation procedure. The chemiluminescence (ECL) intensity of S-g-C_3_N_4_ is five times that of pure g-C_3_N_4_. S-g-C_3_N_4_ forms compounds with Cu^2+^, which quenches its ECL intensity. The addition of L-cysteine improves the performance of the g-C_3_N_4_-based sensor in terms of performance, friendliness of manufacture, ease of implementation, and speed of response due to the increased coordination between Cu^2+^ and L-cysteine. To create an electrochemiluminescence biosensor, cyclodextrin was added to g-C_3_N_4_ for the detection of organophosphate insecticides. The biosensor responds quickly and is highly sensitive. In addition to its application in fluorescent biosensing, g-C_3_N_4_ is also employed in electrochemiluminescent immunosensors. For instance, gold nanoparticles can add new functions to the g-C_3_N_4_ nanosheet to fabricate the immunosensor for the detection of carcinoembryonic antigens [158]. Its high stability and the role of gold nanoparticles can enhance the trapping as well as storing of electrons, which can improve sensing. It exhibits a linear range from 0.02 to 80 ng mL^−1^, and a detection limit of 6.8 pg mL^−1^. There are some more related studies are also present in Figure 4 [155,159,160]. Figure 4a shows the schematic representation of WS_2_ and MoS_2_ biosensor approach. Figure 4b shows the quenching of flu-orescence intensity of FAM-POG and FAM-PRG in the presence (red) and absence (black) of WS_2_ [159]. Figure 4c indicates the mechanism of the Fluorosen-sor-based g-C_3_N_4_ for the detection of Cu^2+^. Figure 4d describes the various concentrations of Cu^2+^ under irradiation in the presence of sunlight, and UV light [155]. Figure 4e presents the schematic illustration of the PEC immunosensor synthesis steps. Figure 4f demonstrates the immunosensor stability of 550 s under irradiation on/off condition for selectivity of the PEC immunosensor [160].

### 4.2. Graphene

**Figure 5:**
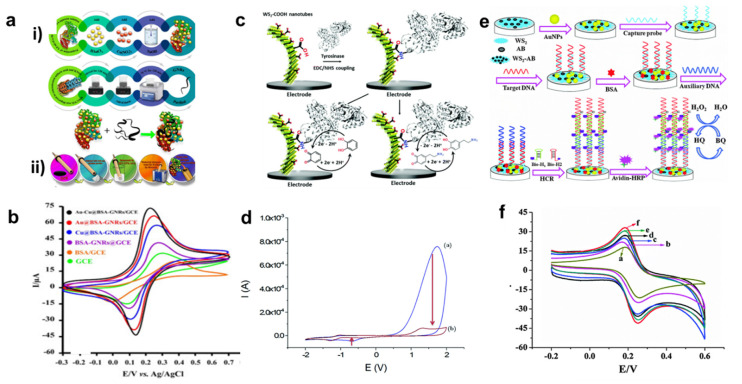
(**a**) Preparation of (**i**) bare GNR, Au-Cu@BSA, and Au-Cu@BSA-GNRs (**ii**) Au-Cu@BSA-GNRs nanocomposite on GCE. (**b**) CV analysis of bare and composite with scan rate of 100 mV/s [162], copyright 2019, Elsevier. (**c**) Sensing of catechol (bottom left) and dopamine (bottom right) using WS_2_ with tyrosinase. (**d**) CV of GCE/WS_2_-COOH electrode (a) first cycle (b) second cycle [168], copyright 2020, Royal Society of Chemistry (**e**) Mechanism involved for detection of DNA using WS_2_ (**f**) CV behavior of WS_2_ and composites with ratio of (a) 3:1 (b), 2:1 (c), 1.5:1 (d), 0.5:1 (e), 1:1 [169], copyright 2016, Journal of Materials Chemistry B.

As a 2D nanomaterial, graphene shows promise as a sensing medium. It has a high surface area, is thermally, optically, and electrically conductive, and is mechanically strong [161]. Graphene’s ability to have a specific shape synthesized from scratch makes it a promising material for use in sensing applications, such as the detection of glucose and cholesterol. Just slight shifts in these might create major medical issues. Platinum/rGO/poly(3-aminobenzoic acid) was used in an amperometric biosensor developed by Ounnunkad et al. for the detection of glucose and cholesterol [126]. The biosensor that was created is sensitive, selective, and responsive enough to be used in clinical diagnostics. Bisphenol is a chemical used in the manufacturing of plastics and other industrial products. Bisphenol is harmful to animals and humans in increasing amounts. Bisphenol levels in the environment must be measured. As a result, the electrochemical biosensor has found widespread application due to its low cost, ease of use, and high sensitivity. Its performance can be improved by combining graphene with other nanomaterials to produce composites. If you compare graphene composites to pure graphene, you will see that the latter is vastly superior for biosensing. Electrodes are manufactured using Au-Cu nanoclusters and graphene nanoribbons [162]. Graphene nanosheet-coated AuPd nanoparticles are also used [163]. Research into finding a cure for cancer remains the biggest obstacle. The importance of cancer detection at an early stage cannot be overstated. Using cancer indicators allows for excellent sensitivity with minimal effort. Graphene has been functionalized with other nanoparticles for use as a cancer biomarker, according to a large body of recent research. For instance, Kong et al. reported [164] the creation of an immunosensor for the detection of carcinoembryonic antigens utilizing gold nanoparticle-thionine-rGO. An anti-carcinoembryonic immobilizer was applied after the material was put on a glassy carbon (GC) electrode. Among the tests performed on it include SEM, UV-visible spectroscopy, electrochemical impedance spectroscopy (EIS), and cyclic voltammetry (CV). It has been shown that carcinoembryonic antigen–antibody complex formation reduces peak current in a concentration-dependent manner. An LOD of 4 pg/mL indicates a concentration range of around 10–500 pg/mL. Similar results were achieved using a tri-antibody dual-channel immunosensor to detect carcinoembryonic antigen and nuclear matrix protein 22 (NMP22) [165] produced from graphene that has been doped with sulfur. Both nuclear matrix protein 22 and carcinoembryonic antigen have a detection limit of 25 fg/mL, but the latter requires 30 fg/mL. This biosensor, based on a tri-antibody dual-channel method, has a low detection limit and great stability, similar to the detection of carcinoembryonic antigens were by 2D TiO_2_ nanosheet and carboxylated g-C_3_N_4_. Many biosensors were detected using epitaxial graphene [166]. Graphene with an edge plane flaw that improves detection was created by anodizing epitaxial graphene. It demonstrates oxygen-related flaws that are thought to make for excellent biosensing platforms for the detection of nucleic acid, uric acid, dopamine, and ascorbic acid. Electrochemical impedance spectroscopy was employed for the detection of DNA, wherein varying quantities of graphene sheets are employed [167]. In this study, they looked at how various graphene sheets affected DNA detection. It has been reported that few-layered graphene performs exceptionally well in detection. There are some more related works that are described in Figure 5 [162,168,169]. Figure 5a shows the preparation of (i) bare GNR, Au-Cu@BSA, and Au-Cu@BSA-GNRs (ii) Au-Cu@BSA-GNRs nanocomposite on GCE. Figure 5b presents the CV analysis of bare and composite with scan rate of 100 mV/s [162]. Figure 5c demonstrates the sensing of catechol (bottom left) and dopamine (bottom right) using WS_2_ with tyrosinase. Figure 5d shows the CV results of GCE/WS_2_-COOH electrode for (a) first cycle (b) second cycle [168]. Figure 5e reveals the mechanism involved for detection of DNA using WS_2_. Figure 5f presents the CV behavior of WS_2_ and composites with various ratios of (a) 3:1 (b), 2:1 (c), 1.5:1 (d), 0.5:1 (e), 1:1 [169].

### 4.3. Boron Nitride (BN)

DNA is detected via electrochemical impedance spectroscopy with the help of variable amounts of graphene sheets. Several types of graphene sheet were investigated to see how they affected DNA detection in this investigation. Reportedly, few-layer graphene excels at detection. Humans and ecosystems alike are particularly vulnerable to mercury’s devastating effects [170]. The detection of mercury is critical because it allows for its usage to be restricted or curbed. SAM of 3-aminopropyltriethoxy silane (ATPES) was utilized to modify cubic boron nitride for the immobilization of dansyl chloride. The dansyl chloride bound to the amine-terminated surface produced a bright fluorescence in the sensor (Mercury concentration-dependent dimming of fluorescence). The fluorescence was restored after the produced sample was submerged in 3 M HCl in an ethanol solution for 15 min. Low-temperature combustion synthesis, carbothermal reduction, and nitridation were used to create the h-BN [171]. A vast surface area is displayed by the flake-like structure of the h-BN that is manufactured. In addition, it has active surface groups and a high defect density. CV and differential pulse voltammetry were applied to this flake-shaped hBN-modified GC electrode. These samples are quite good at detecting ascorbic acid, dopamine, and uric acid. The biosensor has a sensitivity of 3.77 M for ascorbic acid, 0.02 M for dopamine, and 0.15 M for uric acid. Moreover, it results in stable and reproducible output with high immunity to interference. For this reason, flake hBN is a promising material for use in biosensors. Polyimide and polyimide boron nitride were used for the creation of dopamine [172]. Polycondensation reaction employing benzophenone tetracarboxylic dianhydride and diamino di-cyclohexyl methane as precursors produced pure polyimide and polyimide boron nitride. The BN percentage in polyimide presented here ranged from 1% to 3% to 5%. Dopamine detection in the presence of electroactive and non-electroactive species was studied by modifying this prepared sample in a GC electrode. Specifically, research demonstrated that nitride played a pivotal function in enhancing dopamine sensing. The PI-5%BN nanocomposite, with its significantly higher BN content, performs exceptionally well as an electrochemical biosensor. That is because boron nitride enhances the material’s porosity, selectivity, and thermal stability. It reveals modest limits of detection for (4 × 10^−8^ µA µM^−1^). A 2D h-BN nanosheet was also used for dopamine detection in previous work [172]. Many materials, including GC, boron-doped diamond (BDD), and screen-printed graphitic electrodes, were used to modify the h-BN nanosheet through drop-casting (SPEs). It has been found that the h-BN nanosheet treated with SPEs had a better electrochemical response and a lower electrochemical oxidation potential than the bare SPEs. When modified by SPEs, h-BN shows promise as a material for electrochemical biosensors. This cutting-edge Feno resonance biosensor was built with a hybrid nanostructure of plasmonic silver films on silicon (h-BN) [173]. The efficient detection of several biomolecules is facilitated by this biosensor. This is because the h-BN nanosheet can improve absorption efficiency by trapping aromatic biological molecules using dipole–dipole adsorption force. When compared to a standard surface plasmon polariton biosensor, the Feno resonance biosensor is 100 times more sensitive to the detection of biomolecules. Detection at extremely low levels is demonstrated well. The electrical insulating properties of h-BN are due to its bandgap of 5–6 eV. Zhu’s team [174] developed a fluorescent and electrochemical biosensor based on boron nitride-gold nanocluster nanocomposites for the detection of interleukin. Infections and tissue damage trigger the release of interleukin-6. Production of interleukin-6, however, needs to be kept under check. Pathological effects on chronic inflammation and autoimmunity are to be expected as a result of overproduction. The gold nanocluster is immobilized by using poly-(diallyldimethylammonium chloride) (PDDA). The resulting composite has outstanding electrochemical and fluorescent capabilities, in addition to strong stability. The signal response is likewise very clear. Because of its high surface area, the PDDA-BN-Au nanocluster composite that resulted was biocompatible and included a high concentration of gold nanoclusters (GNCs). To create PDDA-BN-GNC-Ab2 bioconjugates, it was employed for immobilizing antibody conjugates (Ab2).

### 4.4. Black Phosphorus (BP)

Black phosphorus (BP) has a distinct structure with corrugated planes of phosphorus (P) atoms which are connected by strong intralayer P-P bonding and weak interlayer Van der Waals forces [175]. It possesses high carrier mobility, high transport anisotropy, bio combativity, and layer-dependent bandgap. Due to these several advantages, the BP is widely used for biosensor applications. A 2D BP nanosheet was used as a fluorescent-based biosensor for the detection of miRNA [176]. The BP nanosheets were prepared using the liquid exfoliation method. This biosensor indicated a detection limit of 9.37 nM and a lower response time, and can detect DNA, proteins, and inorganic ions. The BP nanosheet was used as fluorescent quenching material. Similarly, BP nanosheets were used to study the electrochemiluminescence behavior of the luminol-H_2_O_2_ system [177]. BPN shows a quenching effect on luminol in which luminol considers an energy donor and BPN is an energy acceptor. Protamine was introduced to recover the electrochemiluminescence signal. Protamine can bind to the surface by electrostatic interactions so it can block the energy transfer between BPN and luminol. Thus, the signal is restored. This proposed electrochemiluminescence can detect trypsin in serum samples. The surface area of the biosensor is a significant factor. It helps to accumulate a large number of biomolecules and improve performance. In ref. [178] an ultrasensitive plasmonic biosensor was reported using vertically stacked halloysite nanotubes, MoS_2_, and BP atomic layers upon the gold film. This hybrid showed enhanced efficiency and long-term stability. The halloysite nanotube improved surface area and enhanced the surface plasmonic resonance. A BP/MoS_2_ heterostructure can accumulate more electrons. As a result, a fast carrier charge occurs. Moreover, the ultrasensitive plasmonic biosensor also exhibits adjustable detection sensitivity due to the in-plane anisotropy of BP film. This biosensor provided a label-free detection of small biomolecules. The BP nanoparticles were used as fluorescent biosensing for the detection of DNA [179,180]. BP nanoparticles were synthesized from red phosphorus using high-pressure phase transformation. The obtained sample was investigated with the help of XRD, high-resolution transmission electron microscopy (HR-TEM), X-ray photoelectron spectroscopy (XPS), etc. This study suggests that BP nanoparticles can also be used for the detection of nucleic acid. L. Zhou et al. developed a novel fiber-optic biosensor for human neuron-specific enolase (NSE) cancer biomarkers [181]. Neuron-specific enolase is a highly specific biomarker that is found in patients with various tumors such as neuroendocrine tumors, lung cancer, medullary thyroid cancer, carcinoid tumors, endocrine tumors of the pancreas, and melanoma. The detection of neuron-specific enolase (NSE) is crucial because it provides information about the tumor burden, number of metastatic sites, and response to the treatment. The BP nanosheets were bio-functionalized with poly-L-lysine to improve the light-matter interaction. This prepared sample was integrated with tilted fiber grating. The preparation of BP nanosheets was conducted with ultrasonication. Further BP nanosheets were deposited on fiber grating using the in situ layer-by-layer method. This obtained biosensor showed an enhanced sensitivity towards NSE. It also provided 100-fold greater sensitivity compared to GO and gold nanoparticle-based biosensors. By utilizing the excellent properties of BP, a field-effect transistor biosensor was developed which is used for the detection of human immunoglobulin G [182]. To protect BP getting oxidized in an aqueous solution, a dielectric layer was introduced using Al_2_O_3_. This dielectric layer helps for better stability and sensitivity. The surface of BP was functionalized using gold nanoparticles which were conjugated with antibody probes. The suitable antibody binds with the antigen which leads to the change in electrical resistance of BP, which is measured. The produced BP-functionalized device can detect human immunoglobulin with a lower detection limit of 10 ng/mL and a response time in the order of seconds.

Another work reported that exfoliated BP nanosheets were encapsulated using artificial polypeptide polymer which can form micelles [183]. This allows for improved biocompatibility. Transition electron microscopy shows that the BP nanosheet accumulated in the helical cavity of the copolymer, indicating the hydrophobic nature of nanosheets. Photoluminescence spectroscopy results suggest the polymer micelle was quenched when the BP nanosheet was introduced into the polymer helix. This quenching proves that there is an electron transfer between the BP nanosheet and polymer helix. The encapsulation of the BP nanosheet protects it from oxidation and prevents it from losing its electronic properties. This fabricated 2D hybrid is used for sensing applications. It can also be used for imaging infected tissues and for drug delivery purposes. H. Jiang et al. developed a biosensor for the detection of nucleic acids and proteins [184]. In this work, the modified 2D BP had calcium-cation-doped poly-dopamine (PDA) as the electrode. These 2D BP/PDAs can enter into living cells unaided by transfection agents, resist enzymatic hydrolysis, and show high biocompatibility. Without any aid of chemical conjugation, the poly-dopamine provides binding sites to DNA nucleobases and quenches the fluorescence. This biosensor is highly selective and sensitive to proteins, DNA, and mRNA in complex biological samples.

### 4.5. Molybdenum Disulfide (MoS_2_)

The 2D molybdenum disulfide is a typical member of a large class of transition metal dichalcogenides. MoS_2_ received great attention in diverse fields due to its size-dependent bandgap. The Mo and S atoms have strong ionic bonding and the different layers of MoS_2_ experience weak Van der Waals interactions. The bulk MoS_2_ possesses a direct bandgap of 1.8 eV [185]. It shows high electron mobility and an exposed active site. For instance, a fluorescent biosensor was developed using MoS_2_ nanoflakes for the detection of ferrous ions (Fe^2+^) [186]. Fe^2+^ is seen in all living organisms and is used worldwide in agriculture and industry. The fast detection of ferrous ions is crucial, especially in water samples. The MoS_2_ nanoflakes were synthesized by the hydrothermal method which can catalyze H_2_O_2_ -oxidizing O-phenylenediamine (OPD) substrates to produce a highly fluorescent substance, 2,3-diaminophenazine. The developed MoS_2_/OPD/H_2_O_2_ biosensing provides better sensitivity and selectivity to Fe^2+^ with a detection limit of 3.5 nM. The rod-like MoS_2_ nanostructure was reported to immobilize enzyme molecules to fabricate electrochemical glucose sensors [187]. The shape and size of the nanomaterials are significant for biosensing applications. A nanorod-like MoS_2_ nanostructure was synthesized using a simple one-step synthesis route and used to immobilize the enzyme molecules. The rod-like morphology provides a large surface area that leads to enhancements in the detection of glucose. These enzyme molecules loaded in MoS_2_ maintained their native structure and bioactivity. A fabricated electrochemical glucose biosensor indicated a low detection limit of 0.005 mM and high sensitivity of 25.06 ± 0.5 mA M^−1^ cm^−2^. This biosensor demonstrated excellent reproductivity, sensitivity, and stability for glucose. From this an optic biosensor for miRNA21 biomarkers of breast cancer was developed by taking advantage of the photoluminescence of MoS_2_ flakes [188].

Breast cancer is a major concern in women worldwide. The most used biomarker for breast cancer is miRNA21. Since MoS_2_ exhibits a direct bandgap in the visible region and good stability it is a suitable candidate for photoluminescence biosensing. The epitaxial growth of MoS_2_ on sapphire was observed. The formed MoS_2_ nanoflake surface was modified using a thiolated DNA probe (ss-DNA-SH). Furthermore, the surface was hybridized with complementary and non-complementary miRNA21 sequences. The biomarker recognition was conducted through photoluminescence measurement. Modification with the thiolated DNA probe showed an enhancement in photoluminescence from MoS_2_. However, after the recognition assays, the photoluminescence was quenched. Moreover, the developed optic biosensor based on MoS_2_ nanoflakes improved sensitivity and selectivity.

**Figure 6:**
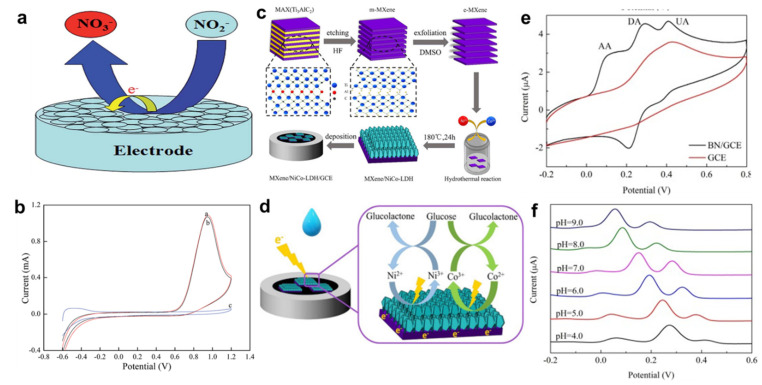
(**a**) Mechanism of nitrite sensor-based h-BN. (**b**) CV analysis of BN whiskers for nitrite sensor with (a) poor crystallized; (b) highly crystalline; and (c) pure Ti electrodes [139], copyright 2016, Royal Society of Chemistry. (**c**) Preparation process involved for MXene/NiCo LDH. (**d**) Proposed mechanism for glucose catalyzing using MXene/NiCo LDH nanocomposite [191], copyright 2019, Elsevier. (**e**) CV analysis of 1000 mM ascorbic acid, 100 mM dopamine, and 400 mM uric acid. (**f**) DPV curves of BN with different pH between 4 to 9 on GCE [171], copyright 2018, Elsevier.

Detection of metal ions found in the environment as well as biological systems has attracted research. Mao et al. used a single layer 2D MoS_2_ as the fluorescence quencher to detect Ag^+^ ion [189]. The detection limit in this assay was 1 nM. Zhang developed a fluorescent biosensor for the detection of uranyl ions (UO_2_^2+^) in the aqueous environment. This sensor was developed based on DNA and MoS_2_. The detection limit in this assay was 2.14 nM [190]. There are some more related studies that are demonstrated in Figure 6 [139,171,191]. Figure 6a shows the mechanism of the nitrite sensor-based h-BN. Figure 6b presents the CV analysis of BN whiskers for nitrite sensor with (a) poor crystallized; (b) highly crystalline; and (c) pure Ti electrodes [139]. Figure 6c describes the preparation process involved for the MXene/NiCo LDH. Figure 6d proposes the mechanism for glucose catalyzing using MXene/NiCo LDH nanocomposite [191] Figure 6e presents the CV analysis of 1000 mM ascorbic acid, 100 mM dopamine, and 400 mM uric acid. Figure 6f shows the DPV curves of BN with different pH between 4 to 9 on GCE [171].

### 4.6. Tungsten Disulfide (WS_2_)

The WS_2_ is composed of a W metal layer sandwiched between two sulfur layers and stacked together by a weak Van der Waals interaction which helps in enhancing planar electric transportation properties [192]. However, WS_2_ suffers from poor electronic conductivity. To overcome this problem WS_2_ is incorporated with good electronic conductivity material to improve its performance. A 2D tungsten disulfide and acetylene black composite were utilized for the electrochemical DNA biosensor [169]. This nanocomposite was synthesized using the hydrothermal method. The sensor was fabricated on gold nanoparticles and a GC electrode was modified by composite. Furthermore, the probe was attached to the modified electrode through the Au-S bond. Afterward, auxiliary DNA was immobilized on the modified electrode. It was then modified with bio-H1-H2 to improve the selectivity of the sensor. The prepared sample indicated a large surface area that can capture more biomolecules, especially DNA. It also reduced the distance for electron transfer and ion diffusion between captured DNA and material. This DNA biosensor conveyed a good linear relationship between the current value and logarithm of target DNA which ranged from 0.001 pM to 100 pM and had a detection limit as low as 0.12 fM. Another work reported an electrochemical biosensor developed by utilizing WS_2_ nanosheets and Poly(indole-6-carboxylic acid) (PIn6COOH) for the detection of the PIK3CA gene in lung cancer [193]. PIn6COOH is a conducting polymer that possesses several properties such as excellent redox activity and excellent stability. This obtained nanocomposite was further modified with immobilization of the probe ssDNA which is achieved by non-covalent p-p stacking. However, the introduction of ssDNA on nanocomposite lead to a decreased current response or signal-off state. When the probe ssDNA was functionalized with target DNA it resulted in the increment of the redox current or signal on the state. It provided a self-signal electrochemical sensing platform, and showed an estimated detection limit of 2.3 × 10^−18^ mol L^−1^ and a dynamic range of 1.0 × 10^−17^ mol L^−1^ to 1.0 × 10^−11^ mol L^−1^ in the detection of a PIK3CA gene related to lung cancer. An amplified photoelectrochemical DNA biosensor was fabricated by making a heterojunction with CdS quantum dots (QDs) and WS_2_ nanosheets, which were further hybridized using chain reaction-mediated enzymatic hydrolysis [194]. Plenty of works have been reported on DNA biosensors by utilizing nanocomposites or hybrids of WS_2_ nanomaterial [195,196].

A novel electrochemical aptamer was reported for the detection of 17b-estradiol using a layered tungsten disulfide nanosheet and gold nanoparticles [197]. An electrochemical aptamer is a biosensor that can generate an electrochemical signal in response to a specific target [198,199,200]. A carboxylic-acid-functionalized WS_2_ nanosheet was used for the immobilization of the tyrosinase enzyme for the detection of catechol and dopamine [168]. This obtained nanotube deposited on GC electrodes showed a satisfactory performance towards the detection of catechol. It indicated a linear range of 0.6–70 µmol L^−1^, a sensitivity of 10.7 ± 0.2 mA L mol^−1,^ and good mass transport. In the case of dopamine detection, it conveyed improved signal capture at a lower concentration. This is due to the electrostatic interaction between the amine function of dopamine and carboxylic acid groups with linear ranges of 0.5–10 µmol L^−1^ and 10–40 µmol L^−1^ and respective sensitivities of 6.2 ± 0.7 mA L mol^−1^ and 3.4 ± 0.4 mA L mol^−1^. An ultrasensitive biosensor was developed for the detection of dopamine by utilizing tungsten disulfide QDs functionalized with GO sheets [201]. The WS_2_ QD shows high luminescence with photoluminescence quantum yield. However, in the presence of graphene oxide, the photoluminescence intensity of WS_2_ QDs was partially quenched. This is due to the Van der Waals interaction and excited charge transfer from WS_2_ QDs to GO. In the presence of dopamine, these prepared GO/WS_2_ QDs nanohybrids were quenched drastically. Thus, selective detection of dopamine was achieved. The morphology of nanostructures has a great impact on the performance of the biosensor. For example, a WS_2_ nanosheet-based fluorescent biosensor was fabricated [202] and was used for the detection of DNA. The 2D WS_2_ nanosheet showed a high quenching ability toward DNA. Peptide nucleic acid is like DNA consisting of normal DNA bases and a peptide-like backbone [203]. PNA has many advantages over DNA. In PNA, the binding affinity and sequence specificity to nucleic acid targets is greater than in DNA [204,205]. Due to its wide advantages, PNA is widely used for biosensing platforms instead of DNA. In this work, a WS_2_ nanosheet was utilized for fluorescent DNA assay by using a more specific PNA probe instead of a DNA probe. Utilizing the WS_2_ nanosheet properties with PNA–DNA hybridization they achieved a simple, fast, stable, and sensitive DNA-detected biosensor through the WS_2_ nanosheets quenching efficiency toward DNA.

### 4.7. MXene

Gogotsi and co-workers created the MXenes in 2011 [206]. It is a new type of 2D nanomaterial that has the potential to boost sensing efficiency. It has many advantages, such as being hydrophilic, electrically conductive, biocompatible, and simple to modify. It is highly stable, has high electrical conductivity, a big surface area, and is easily tunable [207,208,209,210,211,212]. Graphene-like 2D MXene-Ti_3_C_2_ was used to create a nitrite biosensor. Hemoglobin was immobilized on this substance to create a biosensor that does not require a mediator [213]. The acquired material was analyzed in a number of ways to determine its morphology and structure. The data demonstrate the high stability and biocompatibility of MXene-Ti_3_C_2_. A large surface area and strong conductivity make it simple to allow direct electron transmission of hemoglobin. As a result, the constructed biosensor had high nitrite-detection activity over a linear concentration range of 0.5–11,800 µM. Moreover, it had a low detection limit of 0.12 µM. In order to detect cancer biomarkers, researchers produced ultrathin Ti_3_C_2_-MXene nanosheets and bio-functionalized them using amino silane [214]. In a linear detection range of 0.0001–2000 ngmL^−1^, it demonstrated a sensitivity of 37.9 µAng^−1^mLcm^−2^. In order to develop an electrochemical biosensor for the detection of H_2_O_2_, Kai et al. [215] treated the MXene surface with horseradish peroxidase (HRP). An MXene/chitosan/GCE electrode was used to immobilize the HRP. It is clear that this produced electrode exhibited strong activity toward the elimination of H_2_O_2_. The linear range it demonstrated was from 5 to 1650 µmol L^−1^. MXenes were also used in immunosensors, which was described in another study. Immunosensors utilize a variety of antibody and antigen-based immunochemical reactions. Bioanalysis, low cost, small reagent and sample volume, high specificity, and sensitivity are only a few of its many benefits [216]. These high qualities have led to its employment in a wide range of fields, including clinical medicine [217,218], evaluation of environmental pollutants [219,220,221], and many more. A carcinoembryonic antigen immunosensor was developed by Kumar et al. [149]; this antigen is a key cancer biomarker detected in individuals with liver, breast, lung, colorectal, ovarian, and pancreatic cancers. Ti_3_C_2_-MXene functionalized with amino was employed to chemically immobilize COOH terminated-CEA during sensor fabrication. The MXene nanoflakes presented here were created using a layer-delamination synthesis strategy. The biosensor also demonstrated a sensitivity of 37.9 µA ng^−1^ mL cm^−2^ and a linear detection range of 0.0001 to 2000 ng mL^−1^.

### 4.8. Janus Nanoparticles (JNPs)

**Figure 7:**
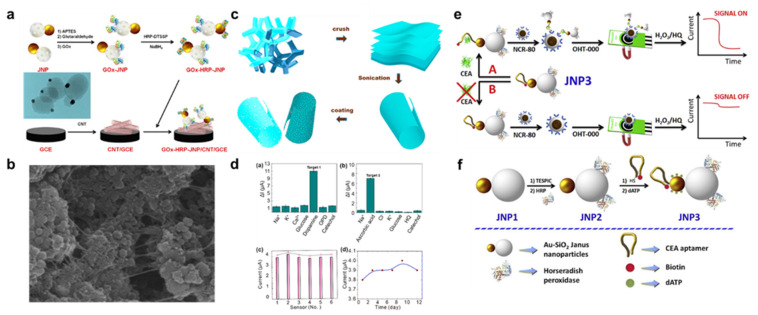
(**a**) Process involved for synthesis of GO/JNP/CNT on GCE. (**b**) FESEM photographs of GO/JNP/CNT [224], copyright 2015, John Wiley and Sons. (**c**) Synthesis of microtube. (**d**) A selective response assessment of a microtube toward (**a**) 5 μM dopamine, (**b**) 20 μM ascorbic acid, (**c**) sensor reproducibility, and (**d**) an aging analysis with 5 μM of ascorbic acid for 1 month [226], copyright 2020, American Chemical Society. (**e**) Display biosensing using Janus nanoparticles. (**f**) Synthesis of aptamer and HRP Janus nanoparticles [227], copyright 2019, Elsevier.

JNPs are a fascinating new class of nanomaterials that show tremendous potential due to their novel traits and versatile applicability. Innovative types of 2DMs called Janus 2DMs have one of the two surfaces functionalized differently or oriented toward a different local environment [222]. The structure of JNPs is amphiphilic. Because of their anisotropic structure, which includes optical, electrical, and magnetic properties, among other things, JPs may have advantages such as adaptability and versatility. Due to their high conductivity and photo/electrothermal or moist sensitivity, carbon-based Janus films can be used as a foundation for the creation of mechanical sensing and actuation technologies [223]. Janus structures are notable for their capacity to engage with their surroundings in a selective manner. Two parts of the structure have different traits, which causes selective interaction to happen. Sometimes a Janus structure can have both a hydrophobic and a hydrophilic side. The structure can then be placed to interact with water in the way that is preferred. A report stated that a composite material made of single-walled carbon nanotubes and gold-mesoporous silica Janus nanoparticles (JNPs) can be used to create biosensors [224]. The use of CNT in this application is advantageous because of its exceptional electrocatalytic, conductive, and matrix-forming 3D transducer properties [225]. The next step was to create an electrochemical biosensor for D-glucose using biofunctionalized JNPs as the active components. Here, glucose oxidase and HRP were immobilized to create a glucose biosensor. By exposing a D-glucose biosensor to different sugars and acids, including D-galactose, D-sucrose, L-arabinose, D-fructose, and ascorbic acid, its selectivity was examined. This biosensor turned out to have a high degree of sensitivity. Using the composite nanomaterials, a highly sensitive, operationally stable, and detection-limit-lowering biosensor was created. Dopamine and ascorbic acid, two biomolecules with crucial physiological roles in humans, are retained in the body at extremely low concentrations [226]. Therefore, ultra-quick diagnosis is essential for modern medical efficacy. The importance of selective electrochemical detection of dopamine and ascorbic acid is highlighted by its application to problems associated with neurological disorders, such as Parkinson’s disease and Alzheimer’s disease. In a report [226], the use of Janus porous nanomembranes for the electrochemical sensing of dopamine and ascorbic acid is described. Here, metal oxide microtubes were made via sonication and atomic layer deposition. Janus pores in nanomembranes can be assisted by atomic layer deposition techniques. On a polyurethane crucible, TiO_2_, ZnO, and Al_2_O_3_ nanomembranes were cultivated. Using ultrasonication, the planar configuration can be transformed into a microtubular one. These sensors’ wide detection range and low detection threshold make them perfect for monitoring dopamine and ascorbic acid levels. These sensors’ wide detection range and low detection threshold make them perfect for monitoring dopamine and ascorbic acid levels. Even after 12 days, the sensor’s performance did not degrade, a testament to the structure’s uniqueness and the porosity’s contribution of additional active sites for sensing across a broad detection range of 0.4–80 mM. For the detection of carcinoembryonic antigens, G. Paniagua et al. developed an amperometric aptasensor using Janus-type nanoparticles with Au and silica faces on opposite ends as an integrated electrochemical biorecognition-signaling system [227]. In this study, both thiolated aptamers and HRP were used to functionalize surfaces in this study. The authors show that the linear detection range of the sensor is between 0.1 pg/mL and 100 ng/mL, with a detection limit of 0.043 pg/mL. The sensor also demonstrated high selectivity, with only a small amount of background noise from competing molecules in the test solution. It has been reported before [228] that ascorbic acid can be detected using Janus carbon nanocomposite and guest-host molecular chemistry. Repetitive attachment of melamine to highly organized pyrolytic graphite with rGO results in a Janus 2D carbon-based nanocomposite. When it comes to detecting ascorbic acid at concentrations as low as 47 pM, the synthesized Janus carbon nanocomposite demonstrated exceptional sensitivity, stability, durability, and repeatability. Perfect molecular coupling between the host and guest molecules at the lattice resulted in enhanced charge mobility, heterogeneous electron transfer, energy density, and stability, all of which contribute to enhanced sensing. There are some more results that are described in Figure 7 [224,226,227]. Figure 7a shows the process involved for synthesis of GO/JNP/CNT on GCE. Figure 7b indicates the FESEM photographs of GO/JNP/CNT [224], Figure 7c shows the process for synthesis of microtube. Figure 7d studies a selective response assessment of a microtube toward (a) 5 μM dopamine, (b) 20 μM ascorbic acid, (c) sensor reproducibility, and (d) an aging analysis with 5 μM of ascorbic acid for 1 month [226]. Figure 7e displays the biosensing approach by using the Janus nanoparticles. Figure 7f shows the steps for synthesis of aptamer and HRP Janus nanoparticles [227].

## 5. Challenges and Counter Makers

**Figure 8:**
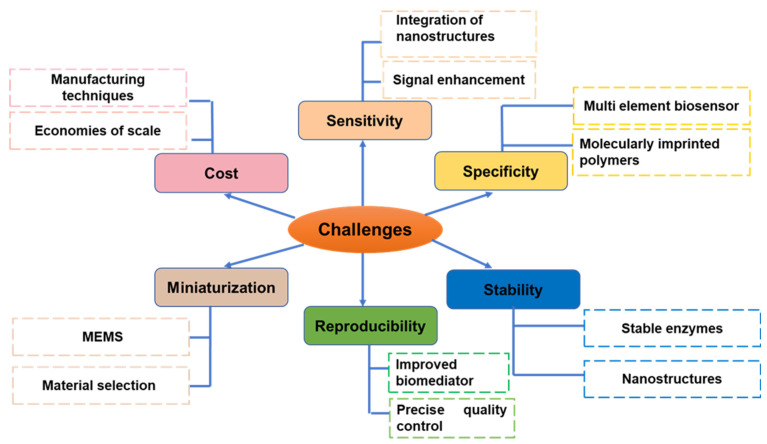
Schematic illustration of possible challenges and future outlooks in the field of biosensor applications.

By utilizing components derived from the organism being studied through analytical signals, biosensors are able to detect and quantify the presence of a target chemical or biomolecule, as well as determine its quantity. In spite of the fact that they offer a number of benefits, including high sensitivity and specificity, as well as real-time monitoring, there are many challenges that prevent their further development and broad application. The development of biosensors is met with a great deal of resistance, but there are also many potential solutions. Figure 8 describes the schematic illustration of possible challenges and future outlooks in the field of biosensor applications. These key points/parameters are discussed in details in the following sections.

**(1) Sensitivity:** A high level of sensitivity is essential for biosensors in order for them to be helpful for detecting trace levels of analytes. Yet, another solution to this issue is to make use of signal amplification approaches such as signal enhancement, signal amplification, and signal transduction. This is just one of several potential solutions. Increasing the selectivity of biosensors can also be accomplished in a number of other ways, including the integration of nanostructures for signal amplification and the use of biological recognition components that are more narrowly focused.

**(2) Specificity:** The degree to which biosensors are able to separate the target analyte from background molecules is a good indicator of their specificity. The employment of many recognition elements by multi-element biosensors allows for the simultaneous detection of a wide variety of analytes, which can contribute to an increase in the sensor’s selectivity. In addition to this, highly specialized molecularly imprinted polymers, also known as MIPs, can be utilized.

**(3) Stability:** For biosensors to be useful over an extended period of time in applications in the real world, they need to be stable. Using nanomaterials that can increase the long-term reliability and stability of biosensors is one alternative that can be utilized. Other solutions include the utilization of stable enzymes and antibodies as well as the utilization of nanostructures that can be utilized.

**(4) Reproducibility:** It is essential for biosensors to have the ability to be replicated in order for them to be commercially successful and extensively used. The use of stringent quality controls at each and every stage of the production process of biosensors is one answer.

**(5) Cost:** The price level at which biosensors are offered may prohibit them from being widely adopted. The production of low-cost biosensors manufactured from readily available materials can be accomplished in one way by employing contemporary manufacturing technologies such as microfabrication and 3D printing.

**(6) Miniaturization:** It is necessary to miniaturize biosensors before they can be helpful in devices that can be held in the hand or that can be worn. Using microelectromechanical systems (MEMS) is one way this can be done. This will allow for the creation of miniature biosensors that can be incorporated into small devices. In the grand scheme of things, taking on these issues will be very necessary for the successful development of biosensors and the broad acceptance of these devices in a variety of disciplines, such as medicine, environmental control, and food standards.

## 6. Conclusions

With their novel characteristics and wide range of potential uses, the growing class of 2D nanomaterials is attracting attention from researchers across disciplines. Even though 2D nanomaterials are currently being studied, more research is still needed. We have reviewed the numerous routes to obtaining 2D nanomaterials through synthesis. Moreover, many types of biosensors make use of various 2D materials. To address these obstacles, researchers in the biosensor sector are performing a number of studies with 2D nanomaterials. Nonetheless, researchers continue to encounter roadblocks. Obtaining 2D nanomaterials with the desired structures and surface functionalization is difficult because no reliable synthesis process exists. It is important to highlight that sensing performances can be enhanced through the selection of precursors and the application of appropriate surface modifications. Long-term stability is a second formidable obstacle. Their structural instability, when they aggregate or collapse, is a critical concern in biosensing applications. The 2D nanomaterial class has vast untapped potential. Appropriate surface functionalization in 2D nanomaterials can circumvent most of the obstacles. Hybridization can only be accomplished by using a superior alternative material. Increased activity in the biosensing platform can be achieved by constructing a homo- or heterojunction. Due to the synergistic impact present in many nanomaterials, hybrid nanomaterials also exhibit certain novel features. There is still extensive research being conducted on the characteristics of 2D nanomaterials and their potential uses in the biosensing sector.

## Abbreviations

Two-Dimensional (2D), graphene oxide (GO), reduced graphene oxide (rGO), graphitic carbon nitride (g-C_3_N_4_), deoxyribonucleic acid (DNA), ribonucleic acid (RNA), Janus nanoparticles (JNPs), quantum dots (QDs), hydrochloric acid (HCl), tetrabutylammonium (NBu_4_)^+^, Janus structures (JPs), Janus two-dimensional materials (2DMs), chemical vapor deposition (CVD), chromium oxide nanoparticles (Cr_2_O_3_ NPs), electro spun carbon nanofiber (ECF), X-Ray diffraction analysis (XRD), photoluminescence (PL), electrochemical impedance spectroscopy (EIS), cyclic voltammetry (CV), scanning electron microscopy (SEM), ultraviolet-visible spectroscopy (UV-Vis), chemiluminescence (ECL), intermolecular charge transfer (ICT), boron-carbon-nitride (BCN), fluorescence resonance energy transfer (FRET), glassy carbon (GC), boron-doped diamond (BDD), poly-(diallyldimethylammonium chloride) (PDDA), gold nanoclusters (GNCs), black phosphorus (BP), uranyl ion (UO_22_^+^), horseradish peroxidase (HRP), hexagonal boron nitride (hBN), transition metal dichalcogenides (TMDCs), high-resolution transmission electron microscopy (HR-TEM), poly-dopamine (PDA), X-ray photoelectron spectroscopy (XPS), neuron-specific enolase (NSE), transition metal oxides (TMOs).
